# Influencing Factors Associated With Mental Health Outcomes Among Dental Medical Staff in Emergency Exposed to Coronavirus Disease 2019: A Multicenter Cross-Sectional Study in China

**DOI:** 10.3389/fpsyt.2021.736172

**Published:** 2021-10-25

**Authors:** Yaopian Chen, Wei Li

**Affiliations:** ^1^Department of Sleep Medicine, Wenzhou Seventh People's Hospital, Wenzhou, China; ^2^Department of Geriatric Psychiatry, Shanghai Mental Health Center, Shanghai Jiao Tong University School of Medicine, Shanghai, China; ^3^Alzheimer's Disease and Related Disorders Center, Shanghai Jiao Tong University, Shanghai, China

**Keywords:** COVID-19, anxiety, depression, stress, dentist, Chinese

## Abstract

**Background:** The epidemic infection of coronavirus disease 2019 (COVID-19) may have a profound impact on dentistry, mainly due to the mode of transmission of the pathogen, which poses a risk to almost all dental operations. Therefore, this study aimed to investigate the prevalence and influencing factors of anxiety, depression, perceived stress, and acute stress disorder among dental medical staff in emergency situations during the COVID-19 epidemic.

**Methods:** From April 3, 2020, to April 10, 20204, a multicenter cross-sectional study was conducted among 808 first-line dental professionals at an emergency department in mainland China. A self-designed questionnaire was used to collect general demographic information. The 7-item Generalized Anxiety Disorder Scale (GAD-7), 9-item Patient Health Questionnaire (PHQ-9), 10-item Perceived Stress Scale (PSS-10), and Acute Stress Disorder Scale (ASDS) were used to assess the severity of symptoms of anxiety, depression, perceived stress, and acute stress disorder (ASD), respectively.

**Results:** The prevalence rates of depression, anxiety, perceived pressure, and ASD among the frontline dental medical staff were 46.4, 36.3, 65.2, and 1.1%, respectively. The frontline dental medical staff who were working in the Wuhan area reported experiencing more anxiety (*p* = 0.038) and perceived stress (*p* < 0.001) compared with those who were not working in the Wuhan area. The frontline dental medical staff who were working in a general hospital reported experiencing more dissociation symptoms (*p* = 0.001) compared with those working in a specialized or private hospital. Individuals with a past medical history reported experiencing more anxiety (*p* = 0.009), depression (*p* < 0.001), and perceived stress (*p* = 0.003) than those without, and individuals with lower levels of education showed higher levels of anxiety (*p* = 0.038). Binary logistic regression analysis results (after controlling for other confounders) suggested that having a past medical history was a risk factor for both anxiety (*p* = 0.002; OR = 2.441; 95% CI, 1.384–4.306) and perceived stress (*p* = 0.001; OR = 1.417; 95% CI, 1.145–1.754).

**Conclusions:** The prevalence of mental symptoms was high among the first-line emergency dental staff. Male sex, working in the Wuhan area, working in a general hospital, a past medical history, and lower levels of education were risk factors. Therefore, we need to pay close attention to the mental health problems of frontline dentists during the COVID-19 outbreak and adopt active preventive strategies to maintain their physical and mental health.

## Introduction

Since the first reported case in December 2019 in Wuhan (China), coronavirus disease 2019 (COVID-19) has widely spread worldwide (1). The World Health Organization declared COVID-19 a pandemic in March 2020, and its reproduction number was significantly larger than 1 (95% CI, 2.24–3.58) (2). As of May 10, 2020, there were 84,431 confirmed cases in China, and 4,643 people died of this viral infection, mainly due to pneumonia and other respiratory complications (3). There is no doubt that frontline doctors and nurses are at greater risk of infection, especially those who are examining and treating patients with COVID-19 (4). The challenges facing healthcare workers include not only the increased workload caused by such outbreaks but also the fear of infection for themselves and their families, the need to use new and often changing protocols, care for patients who are seriously ill, and care for colleagues who are also ill (5). According to a recent report from China, 3.8% of COVID-19 infections occurred among frontline medical staff, of which, 14.8% were classified as severe cases, with a case fatality rate of 0.3% (6, 7). In addition to physical consumption, the psychological burden is a prominent issue for the frontline medical staff (8). Many frontline medical workers have reported feelings of extreme vulnerability, uncertainty, and a threat to life, alongside cognitive and somatic symptoms of anxiety during the early rapid expansion phase of the COVID-19 outbreak (9).

Dental personnel are always at risk of contracting COVID-19 due to close face-to-face contact with patients; frequent use of sharp instruments; and repeated contact with respiratory secretions, blood, saliva, and other contaminated body fluids (10). The possible routes of transmission for COVID-19 in the dental clinic are as follows: contact spread, contaminated surfaces spread, and airborne spread via aerosols formed during dental procedures (11, 12).

Dental staff, who will perform their duties not only in close contact with patients but also while being exposed to droplets and aerosols splashing out of the patient's oral cavity [saliva is one of the main tools of COVID-19 spread (10)], have a high risk of acquiring infection from patients and potentially spreading it to their families, peers, and other patients. Knowing their situation, it is not difficult to understand why dental medical personnel are prone to psychological problems during the epidemic. According to a report by Shacham et al. (13), 11.5% of dental staff showed elevated psychological distress during the COVID-19 outbreak. Moreover, Ahmed et al. (14) found that more than two-thirds (78%) of dentists from 30 countries were scared and anxious about the devastating effects of COVID-19.

However, to the best of our knowledge, there has been no comprehensive evaluation of the psychological impact of COVID-19 on dental medical staff and their mental health conditions in China to date. Therefore, to address this gap in knowledge, this multicenter cross-sectional study investigated the prevalence and influencing factors of anxiety, depression, perceived stress, and acute stress disorder (ASD) among dental medical staff during the COVID-19 epidemic.

## Materials and Methods

### Participants

This national cross-sectional study was conducted in China between April 3, 2020, and April 10, 2020, which was a collaborative effort of 25 dental emergencies located in the eastern, western, and middle parts of China, including Beijing, Shanghai, Gansu, Anhui, and Guangdong. All the questionnaires were collected through the online questionnaire survey platform WeChat (a popular Chinese social media platform) and distributed via social media to solicit participants from the public. Before completing the questionnaire, standardized instructions were presented to the subjects, explaining in detail the content, purpose, and matters needing attention in filling out the questionnaire and emphasizing the principle of voluntary participation and confidentiality of information in the questionnaire. The research subjects were the first-line oral emergency medical staff, who might be engaged in the direct diagnosis, care, and treatment of patients with COVID-19. The inclusion criteria were as follows: (1) age ≥ 18 years, (2) emergency dental staff, and (3) willing to be investigated. The exclusion criteria were as follows: (1) age <18 years, (2) non-dental medical staff or non-emergency, (3) answered a series of questions in the questionnaire identically or in a clear pattern (e.g., choosing the same options), and (4) refusal to be investigated.

**Figure 1:**
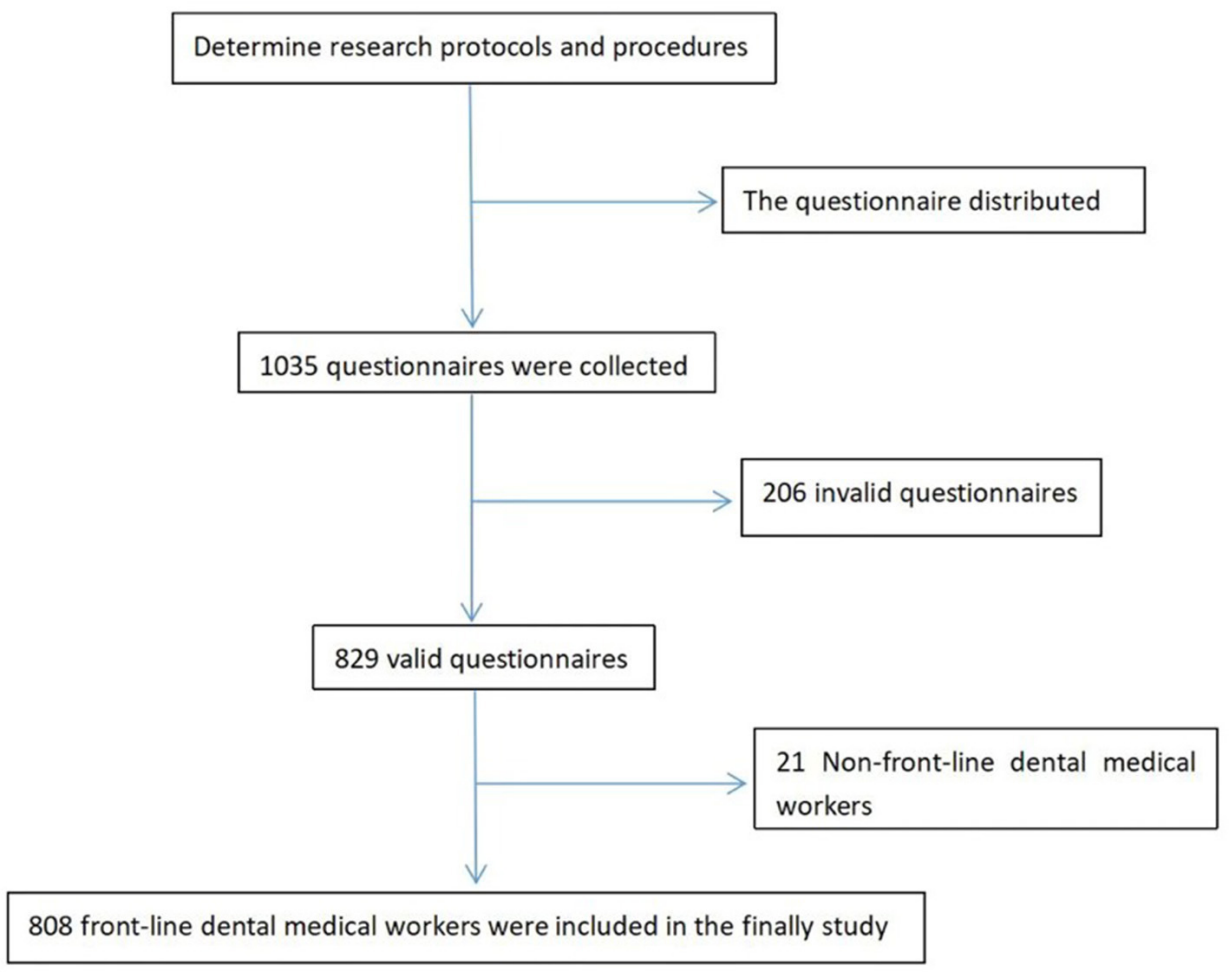
Flow chart of the study.

Collectively, 1,035 first-line oral emergency medical staff across China were enrolled in the study; however, there were 206 invalid questionnaires (e.g., missing information, random filling) and 21 non-dentists. Therefore, 808 people were finally included in the study. The study flow is shown in Figure 1.

Ethical approval was issued by the Ethics Committee of Shanghai Jiao Tong University, and all participants provided informed consent before the study was initiated. The ethical approval number was SH9H-2020-T55-2, and the Chinese Clinical Trial Registration number was ChiCTR2000031538.

### Sample Size

The calculation of the sample size was based on the formula *N* = Zα2P(1 – P)/d2, where Zα = 1.96 and α = 0.05 and the estimated acceptable error range of the scale was 0.1 (15). According to previous studies on the severe acute respiratory syndrome (SARS) outbreak, the proportion of medical staff with comorbidities was estimated to be 50% (16). To facilitate subgroup analysis, we expanded the sample size by 50%, so that at least 800 questionnaires were completed by the participants (17).

### Survey Tools

Using the self-designed questionnaire, we obtained general demographic information (including age, gender, region, marital status, educational level, nature of the hospital, past medical history, and content of worry) about the respondents. The tool used was the Surveystar. As a free questionnaire survey software, the questionnaire star has many powerful functions, such as online voting, online survey, online examination, and intelligent data collection and statistics, and it has been widely used in various scientific research (18–20).

### Neuropsychological Test

The 7-item Generalized Anxiety Disorder Scale (GAD-7) (21), 9-item Patient Health Questionnaire (PHQ-9) (22), 10-item Perceived Stress Scale (PSS-10) (23), and Acute Stress Disorder Scale (ASDS) (24) were used to assess the severity of symptoms of anxiety, depression, perceived stress, and ASD, respectively. The total scores of these neuropsychological tests were as follows: GAD-7, minimal (0–4), mild (5–9), moderate (10–14), and severe (15–21); PHQ-9, no depression (0–4), mild depression (5–9), moderate depression (10–14), moderately severe depression (15–19), and severe depression (20–27); PSS, low stress (0–13), moderate stress (14–26), and severe stress (27 or above); and ASDS, the cutoff value of dissociation cluster was 9, the cutoff value of reexperience, avoidance, and arousal cluster was 28, and the cutoff value of ASD was 56. Therefore, the cutoff scores in this study for detecting symptoms of anxiety, depression, perceived stress, and ASD were 10 (25), 10 (26), 14 (27), and 56 (28), respectively.

#### The GAD-7

The 7-item Generalized Anxiety Disorder Scale (GAD-7) is a self-rating scale for detecting anxiety and has been validated in various populations (29). The GAD-7 is a useful tool to assess anxiety not only in generalized anxiety disorder but also in panic disorder, social anxiety disorder, and post-traumatic stress disorder (PTSD) (30). Previous studies have shown that the GAD-7 has high reliability and validity in the assessment of anxiety symptoms (31).

#### The PHQ-9

The PHQ-9 is a 9-item depression module from the full Patient Health Questionnaire and is designed to screen for the presence and severity of depression. It has been widely used as a measure of mental health well-being and is applicable to patients from different cultures (32). As a screening tool for depressive symptoms, the PHQ-9 scores each criterion from “0” (none at all) to “3” (almost daily), with a total score ranging from 0 (no depressive symptoms) to 27 (all symptoms daily) (26). In the Chinese general population, the Chinese version of the PHQ-9 has been proven to be a reliable and effective screening tool for depression.

#### The PSS-10

The PSS-10 is a self-reported scale to measure the global level of perceived stress. It includes two factors: perceived helplessness and perceived self-efficacy with a score range of 10–50, with a higher score indicating a higher level of perceived stress (33). Previous studies have shown that the PSS-10 exhibits sufficient internal consistency, retest reliability, and validity across different populations (34).

#### The ASDS

The ASDS is a self-report inventory that can be used to assess ASD and PTSD; it is a 19-item inventory that is based on the Diagnostic and Statistical Manual of Mental Disorders (35) criteria, and its sensitivity and specificity are 95 and 83%, respectively (28). The ASDS has shown strong reliability, good sensitivity, and specificity for ASD, such as the COVID-19 outbreak. For example, Lin et al. found that 892 (15.8%) of the first-line hospital workers and first-line management staff had ASD according to the ASDS (36). Zhang et al. used the ASDS to assess acute distress and found that local medical workers had a higher proportion of acute distress than those from outside Hubei (*p* < 0.001) (37). Therefore, we also used the ASDS to assess ASD among first-line medical staff in the department of stomatology during the outbreak. Herein, acute stress events mainly included traumatic reexperience, avoidance and numbness, hypervigilance, and episodic psychotic symptoms.

### Statistical Analysis

Statistical analyses were performed using the Statistical Package for the Social Sciences (IBM SPSS Statistics 22). After scoring the scale score according to standard assignment, the distribution of demographic data and psychological characteristics of all subjects was expressed by frequency and percentage or mean and standard deviation, and the confidence interval was 95%. To determine the impact of different factors on first-line oral emergency medical staff, according to the critical values of the ASDS, PSS-10, PHQ-9, and GAD-7, the respondents were divided into normal, mild, moderate, and severe symptom groups, respectively. The chi-square test was performed for differences in region, nature of the hospital, physical disease, and levels of education. The severity of symptoms between two or more groups was compared using the non-parametric Mann–Whitney *U*-test and Kruskal–Wallis test. Then, based on the results of univariate analysis, the influencing factors with a significance level of <0.05 were selected as independent variables, and the disordered multi-classification variables, such as marital status and unit nature, were set as dummy variables. Binary logistic regression analysis was performed using the results of different scales.

## Results

### General Demographic Data of First-Line Dental Emergency Medical Staff

As described earlier, 808 participants were included in the final study, and their average age was 36.20 ± 8.213 years. Out of the 808 participants, 558 were doctors, accounting for 69.1%, and 250 were nurses, accounting for 30.9%; 6 were in Wuhan, accounting for 0.7%, and 802 were in other areas, accounting for 99.3%; 615 were married (76.1%), 108 were unmarried (22.3), and 13 were divorced or other (1.6%); 385 (47.6%) were in a public general hospital, 405 (50.1%) were in a public specialized hospital, and 18 (2.2%) were in private hospitals; 71 (8.8%) had a history of anxiety or depression; 66 (8.2%) had college degree or below education, 406 (50.2%) had undergraduate education, and 336 (41.6%) had postgraduate and above education; 453 (56.1%) felt that they might come into contact with infected or suspected cases; 650 (80.4%) felt that they might be infected or suspected infected patients in the oral emergency department; 744 (92.1%) believed that there might be asymptomatic infection in patients with oral emergency; 698 (86.4%) believed that COVID-19 was a threat to their or their family's life; 333 (41.2%) felt anxiety, helplessness, or fear of COVID-19; and 48 (5.9%) had participated in the rescue work of SARS or avian influenza. Tables 1, 2 present the results.

**Table 1:** General demographic data of participants.

**Variables**	**No (%)**		
	**Total**	**Occupation**	
		**Physician**	**Nurses**
Overall	808 (100)	558 (69.1)	250 (30.9)
Age, y	36.20 ± 8.213	37.62 ± 8.231	33.05 ± 7.253
**Gender**			
Male, *n* (%)	558 (69.1)	254 (45.5)	6 (2.4)
Female, *n* (%)	250 (30.9)	304 (54.5)	244 (97.6)
**Region**			
Wuhan, *n* (%)	6 (0.7)	2 (0.4)	4 (1.6)
Non-Wuhan, *n* (%)	802 (99.3)	556 (99.6)	246 (98.4)
**Marriage**			
Married, *n* (%)	615 (76.1)	445 (79.7)	170 (68.0)
Unmarried, *n* (%)	180 (22.3)	106 (19.0)	74 (29.6)
Other, *n* (%)	13 (1.6)	7 (1.3)	6 (2.4)
**Nature of the hospital**			
Public general hospital, *n* (%)	385 (47.6)	312 (55.9)	73 (29.2)
Public specialized hospital, *n* (%)	405 (50.1)	228 (40.9)	177 (70.8)
Private hospitals, *n* (%)	18 (2.2)	18 (3.2)	0
**Past medical history**			
Yes, *n* (%)	71 (8.8)	52 (9.3)	19 (7.6)
No, *n* (%)	737 (91.2)	506 (90.7)	231 (92.4)
**Level of education**			
College degree or below, *n* (%)	66 (8.2)	25 (4.5)	41 (16.4)
Undergraduate, *n* (%)	406 (50.2)	204 (36.6)	202 (80.8)
Postgraduate and above, *n* (%)	336 (41.6)	329 (59.0)	7 (2.8)

**Table 2:** Attitudes and views of first-line oral emergency medical staff on COVID-19.

**Variables**	**No, (%)**		
	**Total** **(*n* = 808)**	**Occupation**	
		**Physician** **(*n* = 558)**	**Nurses** **(*n* = 250)**
**Possible direct contact with a confirmed or suspected person**			
Yes, *n* (%)	453 (56.1)	311 (55.7)	142 (56.8)
**There may be infected or suspected infected patients in the**			
**oral emergency department**			
Yes, *n* (%)	650 (80.4)	444 (79.6)	206 (82.4)
**There may be asymptomatic infection in patients with oral emergency**			
Yes, *n* (%)	744 (92.1)	513 (91.9)	231 (92.4)
**Believe that COVID-19 is a threat to your or your family's life**			
Yes, *n* (%)	698 (86.4)	474 (84.9)	224 (89.6)
**Anxiety, helplessness, or fear of COVID-19**			
Yes, *n* (%)	333 (41.2)	224 (40.1)	109 (43.6)
**Participated in the rescue work of SARS or avian influenza**			
Yes, *n* (%)	48 (5.9)	31 (5.6)	17 (6.8)

### Prevalence and Influencing Factors of Anxiety, Depression, and Stress Symptoms

Of the 808 participants, 375 (46.4%) showed depression, 294 (36.3%) showed anxiety, 572 (65.2%) showed moderate or higher perceived pressure, and 9 (1.1%) showed ASD. The participants who were working in the Wuhan area reported experiencing more anxiety (*p* = 0.038) and perceived stress (*p* < 0.001) than those who were not working in the Wuhan area. Those working in a general hospital reported experiencing more dissociation symptoms (*p* = 0.001) than those working in a specialized or private hospital. Individuals with a past medical history reported experiencing more anxiety (*p* = 0.009), depression (*p* < 0.001), and perceived stress (*p* = 0.003) than those without, and individuals with lower levels of education showed higher levels of anxiety (*p* = 0.038). Tables 3, 4 present the results. Next, we compared the total scores of the PHQ-9, GAD-7, PSS-10, and ASDS among different subgroups (region, physical disease, nature of the hospital, and levels of education) with a *post-hoc* examination for multiple comparisons. Finally, we found that employees working in general hospitals had higher PSS scores (*p* = 0.006) and higher ASDS scores (*p* < 0.001) than those working in specialized hospitals, while those with an undergraduate degree had significantly higher PSS scores (*p* = 0.003) than those with a postgraduate degree or higher. Tables 5, 6 present the results.

**Table 3:** Severity categories of anxiety, depression, PSS, and ASDS measurements in total Cohort and subgroups.

**Severity** **category**	**Total, No, (%)**	**Region**			**Nature of the hospital**			
		**No. (%)**		***p***	**No. (%)**			***p***
		**Wuhan**	**Non-Wuhan**		**General** **hospital**	**Specialized** **hospital**	**Private** **hospital**	
**PHQ9, Depression**								
Normal	433 (53.6)	4 (66.7)	429 (53.5)	0.902	193 (50.1)	231 (57.0)	9 (50.0)	0.536
Mild	260 (32.2)	1 (16.7)	259 (32.3)		132 (34.3)	121 (29.9)	7 (38.9)	
Moderate	87 (10.8)	1 (16.7)	86 (10.7)		45 (11.7)	41 (10.1)	1 (5.6)	
Moderate to severe	20 (2.5)	0	20 (2.5)		12 (3.1)	7 (1.7)	1 (5.6)	
Severe	8 (1.0)	0	8 (1.0)		3 (0.8)	5 (1.2)	0	
**GAD-7, Anxiety**								
Normal	514 (63.6)	4 (66.7)	510 (63.6)	0.038^*^	233 (60.5)	269 (66.4)	12 (66.7)	0.645
Mild	233 (28.8)	1 (16.7)	232 (28.9)		118 (30.6)	110 (27.2)	5 (27.8)	
Moderate	47 (5.8)	0	47 (5.9)		25 (6.5)	21 (5.2)	1 (5.6)	
Severe	14 (1.7)	1 (16.7)	13 (1.6)		9 (2.3)	5 (1.2)	0	
**PSS, Perceived stress**								
Mild	281 (34.8)	3 (50.0)	278 (34.7)	<0.001^*^	117 (30.4)	159 (39.3)	5 (27.8)	0.094
Moderate	521 (64.5)	2 (33.3)	519 (64.7)		264 (68.6)	244 (60.2)	13 (72.2)	
Severe	6 (0.7)	1 (16.7)	5 (0.6)		4 (1.0)	2(0.5)	0	
**ASDS, Acute stress**								
Dissociation
Yes	322 (39.9)	3 (50.0)	319 (39.8)	0.687	179 (46.5)	137 (33.8)	6 (33.3)	0.001^*^
No	486 (60.1)	3 (50.0)	483 (60.2)		206 (53.5)	268 (66.2)	12 (66.7)	
**Reexperience**								
No	808 (100)	6 (100)	802 (100)	–	385 (100)	405 (100)	18 (100)	–
**Avoidance**								
No	808 (100)	6 (100)	802 (100)	–	385 (100)	405 (100)	18 (100)	–
**Arousal**								
Yes	7 (0.9)	1 (16.7)	6 (0.8)	0.052	3 (0.8)	4 (1.0)	0	0.870
No	786 (99.1)	5 (83.3)	781 (99.2)		378 (99.2)	390 (99.0)	18 (100)	
**Acute stress disorder**								
Yes	9 (1.1)	0	9 (1.1)	1.000	6 (1.6)	2 (0.5)	1 (5.6)	0.070
No	799 (98.9)	6 (100.0)	793 (98.9)		379 (98.4)	403 (99.5)	17 (2.1)	

*PQH-9, Patien Health Questionnare-9; GAD-7, Generalized Anxiety Disorder-7; PSS, Perceived Stress Scale; ASDS, Acute Stress Disorder Scale*.

* *Means p < 0.05*.

**Table 4:** Severity categories of anxiety, depression, PSS, and ASDS measurements in total Cohort and subgroups.

**Severity** **category**	**Total, No, (%)**	**Physical disease**			**Levels of education**		
		**No. (%)**		***p***	**No. (%)**			***p***
		**Yes**	**No**		**College degree** **or below**,	**Undergraduate**	**Postgraduate** **and above**	
**PHQ9, Depression**								
Normal	433 (53.6)	21 (29.6)	412 (55.9)	<0.001^*^	33 (50.0)	219 (53.9)	181 (53.9)	0.037^*^
Mild	260 (32.2)	31 (43.7)	229 (31.1)		22 (33.3)	133 (32.8)	105 (31.2)	
Moderate	87 (10.8)	13 (18.3)	74 (10.0)		5 (7.6)	45 (11.1)	37 (11.0)	
Moderate to severe	20 (2.5)	5 (7.0)	15 (2.0)		3 (4.5)	5 (1.2)	12 (3.6)	
Severe	8 (1.0)	1 (1.4)	7 (0.9)		3 (4.5)	4 (1.0)	1 (0.3)	
**GAD-7, Anxiety**								
Normal	514 (63.6)	31 (43.7)	483 (65.5)	0.002^*^	40 (60.6)	261 (64.3)	213 (63.4)	0.704
Mild	233 (28.8)	32 (45.1)	201 (27.3)		19 (28.8)	120 (29.6)	94 (28.0)	
Moderate	47 (5.8)	5 (7.0)	42 (5.7)		5 (7.6)	21 (5.2)	21 (6.2)	
Severe	14 (1.7)	3 (4.2)	11 (1.5)		2 (3.0)	4 (1.0)	8 (2.4)	
**PSS, perceived stress**								
Mild	281 (34.8)	12 (16.9)	269 (36.5)	0.004^*^	21 (31.8)	126 (31.0)	134 (39.9)	0.100
Moderate	521 (64.5)	58 (81.7)	463 (62.8)		44 (66.7)	278 (68.5)	199 (59.2)	
Severe	6 (0.7)	1 (1.4)	5 (0.7)		1 (1.5)	2 (0.5)	3 (0.9)	
**ASDS, acute stress**								
**Dissociation**								
Yes	322 (39.9)	41 (57.7)	281 (38.1)	0.001^*^	27 (40.9)	166 (40.9)	129 (38.4)	0.775
No	486 (60.1)	30 (42.3)	456 (61.9)		39 (59.1)	240 (59.1)	207 (61.6)	
**Reexperience**								
No	808 (100)	71 (100)	737 (100)	–	66 (100)	406 (100)	336 (100)	–
**Avoidance**								
No	808 (100)	71 (100)	737 (100)	–	66 (100)	406 (100)	336 (100)	–
**Arousal**								
Yes	7 (0.9)	0	7 (1.0)	1.000	1 (1.5)	3 (0.7)	3 (0.9)	0.821
No	786 (99.1)	70 (100)	716 (99.0)		65 (98.5)	399 (99.3)	322 (99.1)	
**Acute stress disorder**								
Yes	9 (1.1)	1 (1.4)	8 (1.1)	0.565	1 (1.5)	4 (1.0)	4 (1.2)	0.916
No	799 (98.9)	70 (98.6)	729 (98.9)		65 (98.5)	402 (99.0)	332 (98.8)	

*PQH-9, Patien Health Questionnare-9; GAD-7, Generalized Anxiety Disorder-7; PSS, Perceived Stress Scale; ASDS, Acute Stress Disorder Scale*.

* *Means p < 0.05*.

**Table 5:** Severity of anxiety, depression, PSS, and ASDS measurements in different subgroups.

**Neuropsychological** **tests**	**Regions**				**Nature of the hospital**				
	**Wuhan**	**Non-wuhan**	**Statistics**	***P*-value**	**General**	**Specialized**	**Private**	**Statistics**	***P*-value**
PHQ9, Depression	4.50 ± 4.506	4.84 ± 4.478	−0.172	0.863	5.18 ± 4.537	4.50 ± 4.403	5.28 ± 4.430	5.910	0.052
GAD-7, Anxiety	4.67 ± 7.367	3.62 ± 3.927	−0.358	0.720	3.89 ± 4.046	3.35 ± 3.867	4.17 ± 3.761	4.910	0.086
PSS, perceived stress	14.83 ± 8.589	15.18 ± 4.979	−0.684	0.494	15.69 ± 4.869	14.65 ± 5.127	16.22 ± 4.008	7.909	0.019^*^
ASDS, acute stress	42.50 ± 21.879	34.32 ± 12.279	−0.801	0.423	35.87 ± 12.566	32.91 ± 11.900	35.61 ± 12.236	16.355	<0.001^*^
**Neuropsychological** **tests**	**Physical disease**				**Levels of education**				
	**Yes**	**No**	**Statistics**	***P*** **-value**	**College degree** **or below**,	**Undergraduate**	**Postgraduate** **and above**	**Statistics**	***P*** **-value**
PHQ9, Depression	7.20 ± 4.732	4.61 ± 4.387	−4.806	<0.001^*^	5.70 ± 5.668	4.71 ± 4.235	4.83 ± 4.491	0.615	0.735
GAD−7, Anxiety	5.32 ± 4.355	3.46 ± 3.880	−3.906	<0.001^*^	4.24 ± 4.601	3.40 ± 3.564	3.77 ± 4.253	1.229	0.541
PSS, Perceived Stress	17.27 ± 4.276	14.98 ± 5.029	−3.681	<0.001^*^	16.14 ± 5.285	15.52 ± 4.683	14.58 ± 5.270	10.973	0.004^*^
ASDS, Acute Stress	40.15 ± 12.651	33.82 ± 12.212	−4.515	<0.001^*^	35.32 ± 11.828	34.66 ± 12.391	33.85 ± 12.470	2.305	0.316

*PQH-9, Patien Health Questionnare-9; GAD-7, Generalized Anxiety Disorder-7; PSS, Perceived Stress Scale; ASDS, Acute Stress Disorder Scale*.

* *Means p < 0.05*.

**Table 6:** *Post-hoc* examination for multiple comparison.

**Neuropsychological tests**	**Group 1**	**Group 2**	**Statistics**	***p***
PSS, perceived stress	General hospital	Specialized hospital	7.505	0.006^*^
		Private hospital	0.009	0.924
	Specialized hospital	Private hospital	1.089	0.297
ASDS, acute stress	General hospital	Specialized hospital	16.415	<0.001^*^
		Private hospital	0.309	0.578
	Specialized hospital	Private hospital	0.293	0.588
PSS, perceived stress	College degree or below	Undergraduate	0.881	0.348
		Postgraduate and above	4.153	0.125
	Undergraduate	Postgraduate and above	8.687	0.003^*^

*PQH-9, Patien Health Questionnare-9; GAD-7, Generalized Anxiety Disorder-7; PSS, Perceived Stress Scale; ASDS, Acute Stress Disorder Scale*.

* *Means p < 0.05*.

### Risk Factors for Anxiety, Depression, and Stress Symptoms

The results of binary logistic regression analysis (after controlling for other confounders) suggested that having a past medical history was a risk factor for both depression (*p* = 0.002; OR, 2.441; 95% CI, 1.384–4.306) (the total score of PHQ-9 was 10 as the threshold to determine whether the subjects had depression, and then it was taken as the dependent variable. Independent variables included history of physical illness and years of education; *R* = 0.01; *R*^2^ = 0.019; the *p*-value of the Hosmer–Lemeshow test was 0.709) and perceived stress (*p* = 0.001; OR = 1.417; 95% CI, 1.145–1.754) (the total PSS score was 14 as the threshold to determine whether the subjects had perceived pressure, and then it was taken as the dependent variable. Independent variables included work location, history of physical illness, and years of education; *R* = 0.016; *R*^2^ = 0.022; the *p*-value of the Hosmer–Lemeshow test was 0.731) (since we did not find meaningful factors based on the GAD-7 and ASDS thresholds, we did not present these two parts of the data). Tables 7, 8 present the results.

**Table 7:** Risk factors for mental health outcomes identified by binary logistic regression analysis (depression).

**Variables**	**B**	**S.E**.	**Wals**	**df**	***P***	**OR**	**95%confidence** **interval**
Past medical history	0.892	0.290	9.498	1	0.002^*^	2.441	1.384~4.306
Levels of education	0.002	0.165	0.018	1	0.894	1.022	0.740~1.411

* *Means p < 0.05*.

**Table 8:** Risk factors for mental health outcomes identified by binary logistic regression analysis (PSS).

**Variables**	**B**	**S.E**.	**Wals**	**df**	***P***	**OR**	**95%confidence** **interval**
Past medical history	0.349	0.109	10.287	1	0.001	1.417	1.145~1.754
region	0.725	0.835	0.754	1	0.385	2.064	0.402~10.600

*Means p < 0.05.

## Discussion

To the best of our knowledge, this is the first multicenter cross-sectional study to explore the influence of COVID-19 on anxiety, depression, and stress among the first-line emergency dental staff in China. We arrived at several interesting conclusions: first, there was a high prevalence of mental symptoms among the first-line emergency dental staff, and the prevalence rates of depression, anxiety, perceived pressure, and PTSD were 46.4, 36.3, 65.2, and 1.1%, respectively. Second, working in the Wuhan area, working in a general hospital, a past medical history, and lower levels of education were associated with more mental symptoms. Third, having a past medical history was a risk factor for both anxiety and perceived stress.

The epidemic COVID-19 infection may have a profound impact on dentistry, mainly due to the mode of transmission of the pathogen, which poses a risk to almost all dental operations (37). As many patients with COVID-19 and asymptomatic (carrier) patients may present for dental treatment in outpatient dental settings or at the emergency department, dentists are included in the highest risk categories for transmission and contraction of COVID-19 (38). In addition to the protective equipment recommended for dentists (e.g., filtering facepiece respirators, protective glasses, or face shields), special attention should be paid to their mental health. Herein, we found that the vast majority of dental practitioners believed that they were coming into contact with confirmed or suspected cases and were worried about passing the virus to their families. In addition, ~50% of all frontline dental workers expressed fear, helplessness, and concern about COVID-19.

Using a series of neuropsychological tests, such as the PHQ-9, GAD-7, PSS-10, and ASDS (the cutoff scores for detecting symptoms of anxiety, depression, perceived stress, and PTSD were 10, 10, 14, and 56, respectively), we found that the prevalence rates of depression, anxiety, perceived pressure, and PTSD among the first-line dental medical staff in the emergency department were 46.4, 36.3, 65.2, and 1.1%, respectively. Before our study, many similar studies (but their study subjects were not emergency dental personnel) were conducted in China. For example, Li et al. (39) found that the prevalence rates of insomnia and general psychological symptoms among home medical staff in Ningbo were 24.97 and 8.64%, respectively. Lu et al. (40) found that frontline medical staff with close contact with infected patients were twice as likely to suffer depression and anxiety and 1.4 times more likely to feel fear compared with the non-clinical staff. Kang et al. (41) found that the prevalence rates of subthreshold, mild, moderate, and severe mental health disturbances among 994 medical and nursing staff working in Wuhan in the immediate wake of the viral epidemic were 36.9, 34.4, 22.4, and 6.2%, respectively. However, due to the inconsistency of the evaluation tools and the difference in survey purposes, we could not judge whether our findings were consistent or not. Nevertheless, we could determine that healthcare workers in first-line emergency dentistry experienced a high psychological burden during the COVID-19 outbreak.

We also investigated the incidence of psychological problems (e.g., anxiety, depression, perceived stress, and PTSD) in different populations and subgroups. Our findings indicated that medical staff working in the Wuhan area reported more severe symptoms of anxiety and perceived stress than those in non-Wuhan areas, which was consistent with the findings of the study by Lai et al. (17). Since Wuhan was the origin and development center of COVID-19, it was not difficult to understand why the medical staff working in Wuhan had more psychological stress and anxiety. We also found that individuals with a past medical history had higher levels of depression, anxiety, perceived stress, and dissociation symptoms, and the results of the binary logistic regression analysis showed that a past medical history was the main risk factor for depression and perceived stress. Previous studies have suggested that poor physical condition is associated with a higher incidence rate of psychological disorders. Another psychological study of COVID-19 in China also showed that poor physical conditions were significantly associated with a higher rate of mental illness (42). Therefore, the conclusions of our study were consistent.

We also found that men working in a general hospital and having lower levels of education were associated with more severe psychopathological symptoms. The link between dissociation and sex has been confirmed in several studies (43–45), and it is generally believed that women are more likely to suffer from dissociation (46); therefore, our conclusions were inconsistent. Since there was no difference between the total scores of males and females in the ASDS scale but only in the dissociation sub-items, it may not have much practical significance. Similarly, the aforementioned conclusion applies to unit properties, as we also found that medical staff working in a general hospital also showed more severe symptoms of dissociation. We speculated that this might be related to the fact that public general hospitals had more patients and, therefore, had a greater risk of infection (47). Interestingly, we found that people with low education tended to have higher levels of anxiety. Since nurses and women make up the majority of those with less education, it was easy to explain why these people were more prone to anxiety.

The strength of our study is that it is the first nationwide study of dental practitioners' psychological responses to COVID-19, and the sample size is advantageous. However, our study has certain limitations: first, it is a cross-sectional study that cannot establish a causal link between COVID-19 and psychopathological symptoms; second, we used the scale score as the basis to assess whether the subjects had psychopathological symptoms, rather than clinical diagnosis, so it may cause some deviation in the results; third, it was unclear whether the psychological or psychiatric conditions of the first-line emergency dental staff might influence their work. Moreover, the mental health status of individuals before enrollment was not considered in our study.

## Conclusions

There was a high prevalence of mental symptoms among the first-line emergency dental staff. Men working in the Wuhan area, working in a general hospital, a past medical history, and lower levels of education might be risk factors. Therefore, we need to pay close attention to the mental health problems of frontline dentists during the COVID-19 outbreak and adopt active preventive strategies to maintain their physical and mental health.

## Data Availability Statement

The original contributions presented in the study are included in the article/supplementary material, further inquiries can be directed to the corresponding author/s.

## Ethics Statement

The studies involving human participants were reviewed and approved by the Ethics Committee of Shanghai Jiao Tong University. The patients/participants provided their written informed consent to participate in this study.

## Author Contributions

YC analyzed the data and drafted the manuscript. WL wrote the manuscript. All authors contributed to the article and approved the submitted version.

## Funding

This work was supported by Grants from the Clinical Research Center Project of Shanghai Mental Health Center (CRC2017ZD02), Cultivation of Multidisciplinary Interdisciplinary Project in Shanghai Jiaotong University (YG2019QNA10), curriculum reform of the Medical College of Shanghai Jiaotong University, and Feixiang Program of Shanghai Mental Health Center (2020-FX-03) and A study on sleep disorders in the elderly in Wenzhou, Wenzhou public Welfare Science and technology (Project no.: Y20170373).

## Conflict of Interest

The authors declare that the research was conducted in the absence of any commercial or financial relationships that could be construed as a potential conflict of interest.

## Publisher's Note

All claims expressed in this article are solely those of the authors and do not necessarily represent those of their affiliated organizations, or those of the publisher, the editors and the reviewers. Any product that may be evaluated in this article, or claim that may be made by its manufacturer, is not guaranteed or endorsed by the publisher.
